# *In Situ* Split of the Liver When Portal Venous Embolization Fails to Induce Hypertrophy: A Report of Two Cases

**DOI:** 10.1155/2013/238675

**Published:** 2013-12-08

**Authors:** Bergthór Björnsson, Thomas Gasslander, Per Sandström

**Affiliations:** ^1^Department of Surgery, County Council of Östergötland, 581 85 Linköping, Sweden; ^2^Division of Surgery, Departmet of Clinical and Experimental Medicine, Faculty of Health Sciences, Linköping University, 581 85 Linköping, Sweden

## Abstract

*Introduction*. Associated liver partition and portal vein ligation for staged hepatectomy (ALPPS) has been reported as an efficient alternative to portal vein embolization (PVE) to induce growth of the future liver remnant (FLR). This method combines portal vein ligation with splitting of the liver parenchyma. Although shown to be efficient in introducing growth of the FLR and allowing for resection of the deportalized part of the liver one to two weeks after the first operation, this approach carries a significant mortality. *Presentation of Case*. ALPPS was applied to two elderly patients where PVE failed to stimulate sufficient growth of the FLR. In both cases, subsequent growth of the FLR allowed for successful resection of the liver lesions. The postoperative course was uneventful for both patients. *Discussion*. In both cases, the growth of the FLR was similar to what was previously reported when ALPPS has been performed, both patients underwent radical resections that would probably not have been safe after only the PVE. *Conclusion*. ALPPS used as rescue technique when PVE fails to stimulate sufficient growth of the FLR can be expected to deliver similar results as ALPPS “Up front”. These cases also suggest that ALPPS is applicable to the elderly population.

## 1. Introduction

Portal vein embolization (PVE) is the main method used to induce growth of the future liver remnant before large liver resections [1]. In addition to PVE, portal vein ligation is an important tool in the arsenal when the distribution of liver tumours alone requires a two-stage approach [2]. Recently ALPPS has been reported to induce accelerated growth of the FLR allowing for two-stage liver resections with short interval between the procedures. ALPPS consists of portal vein ligation and division (*in situ* split) of the liver parenchyma between the surgical specimen and the future liver remnant (FLR) but leaving the liver artery, bile duct, and the liver vein intact until the subsequent operation. At the second operation, (one to two weeks later) the remaining liver artery, bile duct, and liver vein are divided and the surgical specimen was extracted.

This new method, however, implies an additional operation and possibly more morbidity and mortality than PVE followed by one-stage liver resection. In addition, its superiority to PVE has been questioned [3]. So far, only 88 patients have been reported in the six series published on ALPPS and six of those have had ALPPS as a rescue treatment when PVE failed [4–9]. Additionally, there are some reported successful cases of ALPPS, and it has been suggested that this new approach should be reserved as a rescue treatment for failed PVEs.

We present two cases of elderly patients treated primarily with PVE but then operated in two-stage manner with ALPPS as the liver hypertrophy gained with the PVE was insufficient.

## 2. Presentation of Case

### 2.1. Case 1

**Figure 1:**
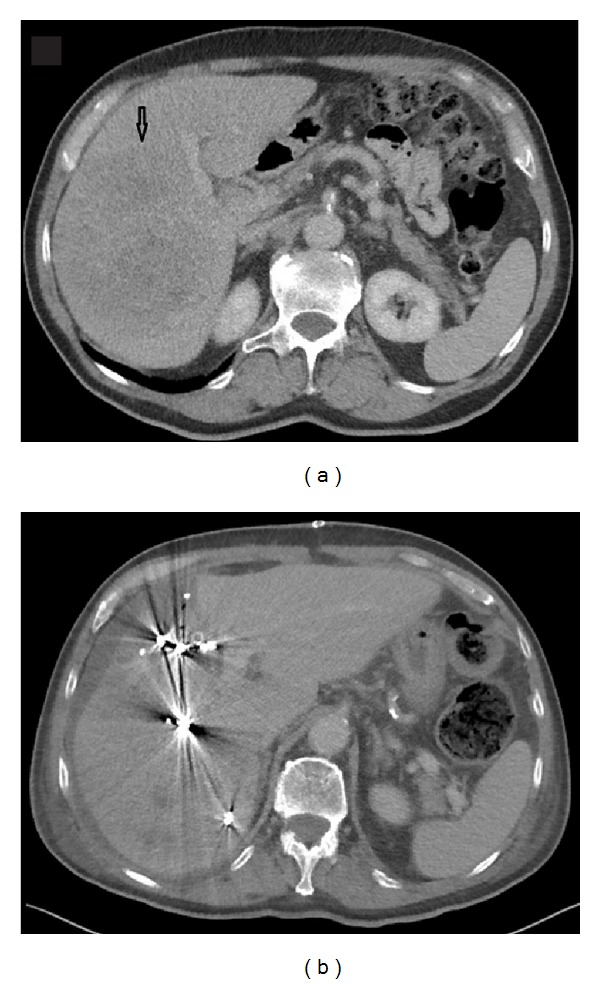
The liver in a patient with a large HCC on the right side before portal venous embolization (a) and the day after ISS (b). The arrow shows the tumor.

A 77-year-old male (72 kg) with no history of liver disease presented with nausea and vomiting. After a gastroscopy without pathological finding, a CT scan revealed a 128 mm tumour in the right liver lobe extending into segments 4A and 4B (Figure 1). The radiological characteristics of the mass were consistent with a hepatocellular carcinoma (HCC). Alpha fetoprotein was elevated to 3150 kU/L. Further radiological workup did not reveal any signs of metastatic disease. The FLR did not show certain radiological signs of cirrhosis and the Child-Pugh score was 5. A multidisciplinary tumour board assessed the mass resectable with a right trisegmentectomy. However, the FLR was found to be insufficient in volume on volumetry done with CT scan (472 mL, <20% of total liver volume). In order to stimulate growth of the FLR, PVE on all right-sided portal vein branches was performed with Bead Blocksphere 100–300 micrometer and coils (Biocompataibles UK Limited, Surrey, UK). At the time of PVE, the risk of accidental embolisation of the portal branches to segments 2 and 3 was found to be high if PVE was to be applied to segment 4.

No complications were noted to the PVE and the patient was discharged. At surgical exploration, the FLR was found insufficient in size for the resection. An *in situ* split was therefore performed just to the right of the falciform ligament. Transection of the liver parenchyma was done using an ultrasonic dissector/aspirator (Cavitron Ultrasonic Surgical Aspirtaor, CUSA System, Valleylab Inc., Boulder CO), bipolar diathermy, clips, and sutures without applying the Pringle manoeuvre. The portal branches to segments 4A and 4B were divided and vessel loops were placed around the right portal pedicle and the right liver vein. Operation time was 295 minutes and estimated blood loss was 2000 mL.

**Figure 2:**
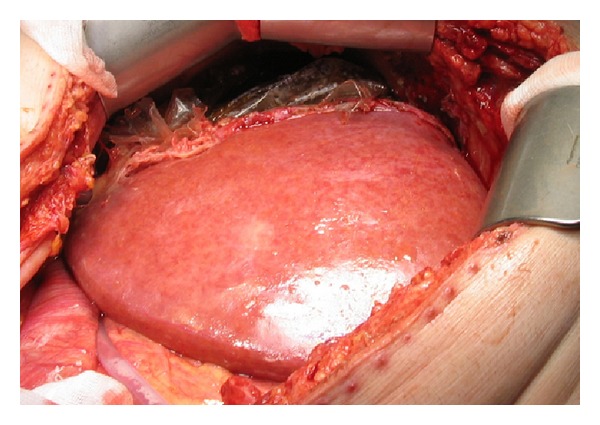
The liver in a patient with large HCC on the right side at the beginning of stage 2 operation; a plastic bag is placed around the right liver half and segment 4.

Seven days after the primary operation, a CT scan revealed growth of the FRL to 973 mL (106%). On the 9th day after the ISS procedure, the second procedure was performed (Figure 2). The right portal pedicle was divided using Endo GIA Ultra Universal with vascular stapler (Covidien, Mansfield, MA, USA), and the same method was used to divide the right and median liver veins. The operation time was 90 minutes and estimated blood loss was 100 mL. Twelve days later, the patient was discharged after an uneventful hospital stay. INR peaked at 1,5 and bilirubin at 54 micromol/L, but both returned to normal before discharge. The pathological examination revealed an 180 mm radically resected HCC without signs of vascular invasion.

### 2.2. Case 2

**Figure 3:**
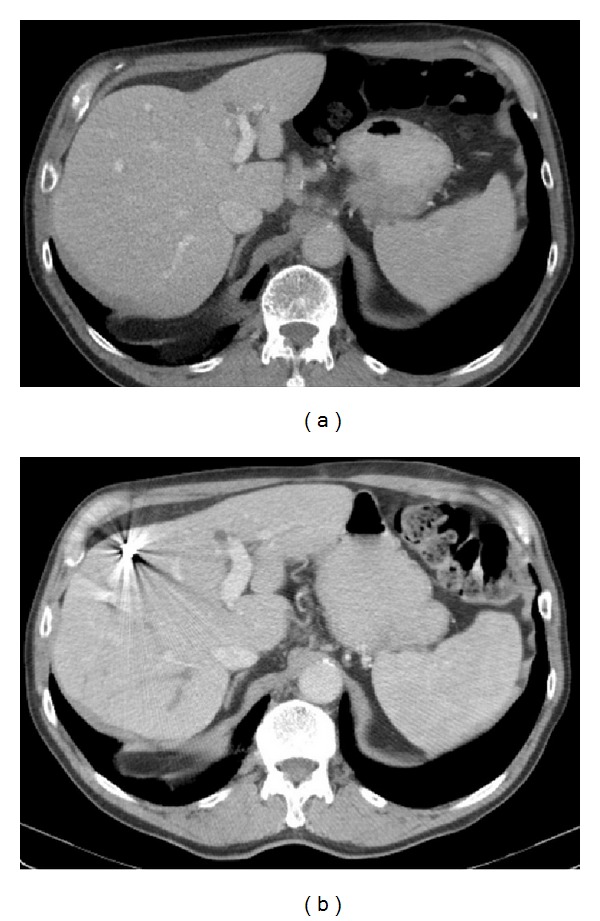
The liver in a patient with a large colorectal liver metastases on the right side before (a) and after (b) portal venous embolization.

**Figure 4:**
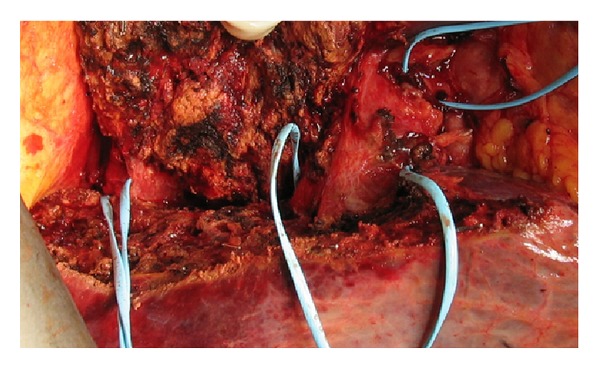
The liver in a patient with large colorectal liver metastases on the right side at the end of stage 1 operation. Ligaloops are placed around the right hepatic vein, the right portal triad, and the right hepatic artery.

An 80-year-old male (77 kg), operated 4 years previously with resection of the sigmoid colon because of a T3N1M0 adenocarcinoma, had been lost to follow up after the colonic surgery. When returning he presented with elevated CEA and a workup including CT scan of the abdomen revealed a 95 mm tumour in the right liver lobe. The tumour was located mainly in segments 5 and 6 (Figure 3), but it was extended towards the portal branches to segments 7 and 8. He was presented at a multidisciplinary tumour board. A decision was made to start chemotherapy in order to downsize the tumour. The left liver lobe was noted to be small (348 mL, <20% of total liver volume). A follow-up CT scan after 2 months of chemotherapy showed little or no reduction in tumour size. The strategy was changed to a preoperative right-sided PVE followed by a right-sided hemihepatectomy. A new CT scan, after PVE, revealed a 42% growth of the FLR, but it was still only 23% of the total liver volume. As the patient had received chemotherapy, this was found to be insufficient for resection. To further stimulate the growth of the FLR, ISS between segment 5/8 and segment 4 was done and vessel loops were placed around the right portal pedicle and the right liver vein at the first operation that lasted 164 minutes with estimated blood loss of 300 mL (Figure 4). A CT scan 6 days later showed 95% growth of the FLR compared to the initial volume and it was now >30% of the total liver volume. On the 7th postoperative day a right-sided liver resection was done in the same manner as described above. That operation took 55 minutes and the estimated blood loss was 200 mL. The postoperative course was uneventful and the patient was discharged on the 10th postoperative day. The pathological examination showed a radically resected adenocarcinoma metastasis.

## 3. Discussion

The novel ALPPS must be considered one of the major progressing techniques within liver surgery for patients with initially unresectable disease. It seems to be an important option in cases with bilobar disease where two-stage surgery is the only option. However, it remains to be seen if this aggressive treatment could completely replace PVE as a method to induce growth of the FLR. It has to be kept in mind that every surgical procedure carries an inherent risk for complications, and thus PVE followed by one-step resection may be an appropriate approach for some patients. In the two cases presented the initial strategy was to carry out a single step resection after PVE. As the PVE showed to be insufficient for these patients, ISS was added as a rescue treatment allowing for successful resections. These findings are similar to the previously reported use of ALPPS as a rescue treatment after failed PVE [5].

## 4. Conclusion

In patients with the need of extensive growth of the FLR, PVE is rarely enough as a preoperative growth stimulation, and the ALPPS concept is the only alternative today. In patients with a need for 10–30% growth of the FLR, PVE may be the best alternative. In cases where the growth still is not enough after PVE, ISS may be added as a rescue option. Further, these cases suggest that the ALPPS concept is also applicable to the elderly population. As there is no reliable method of knowing in beforehand if PVE will induce the growth needed for one-stage resection, ISS can be used as a second-line treatment for the patients that do not respond adequately to PVE. In this way PVE may avoid unnecessary two-stage resections in patients where resections in the FLR are not needed.
